# Foreign Summary

**Published:** 1840-06-20

**Authors:** 


### FOREIGN SUMMARY.

### ANDRAL'S LECTURES ON THE ALTERATIONS OF THE BLOOD.—NO. I.

*General and preliminary observations—Importance of attending to the state of the blood—Nature of the alteration which this fluid undergoes—Such change affects its quantity or its quality—Changes of quantity—Phenomena attending plethora and anemia.*

In those sciences which are called natural there is no primitive fact such as may be found in some others. Geometry is based upon the law of gravitation. In our study of the natural sciences, it is sufficient to analyse the facts, to classify, to bring them together, or separate them, as may be necessary: for this purpose different methods have been employed. Some, supposing the existence of a primitive principle, endeavoured to arrive at it, and sought for it in the material, or the dynamic, state of life. In pathology, it has been sought for in the solids and fluids; at one time, incitability, named also excitability, or the vital forces, was considered as its seat. Others have attempted to study the laws of the vital forces, as they would those of gravitation;—the attempt has been fruitless, for we can find no primitive fact from which the particular ones can be deduced. In the actual state of science, we cannot trace any general fact which will give us a key to all the others, as gravitation gives us the solution of all the facts in geometry.

Many persons at the present day reject any researches on this subject, and merely study the facts themselves; such are the empirics or exclusives, who, seeing the errors of our predecessors, will not inquire into the causes of effects. All those who, at the present day, give themselves up to the study of these sciences, admit a succession of facts drawn from the different systems of fluidism, solidism, &c., and recognise those which have stood the test of ages, while they reject those which are erroneous; and I am willing to admit, that all the doctrines that have swayed the minds of men do contain some one fact or other, although the conclusions drawn from them may have been false. This doctrine is at present much in vogue, and by it we explain one fact by solidism, another by fluidism, and another by vitality. At present every body is eclectic, while twenty years ago it required no small degree of courage to be eclectic, or fluidist, or to maintain that the alterations of the blood had any influence over diseases. I found that the theory of solidism could not explain a great number of facts, and I sought for their solution in the different theories of fluidism, &c., and thus I became eclectic, whilst almost all were still influenced by the doctrines which then prevailed.

We have to study the forces which have been created with matter, and the influence which these forces exercise over it. Are the forces which produce the phenomena in organized bodies identical with those which produce them in unorganized, or bodies invert? Although organized bodies possess functions peculiar to themselves, we are not to conclude from hence that they do not possess properties which belong also to matter in general. We have two orders of phenomena: one produced by laws which regulate matter in general, the other by laws which regulate organized matter. Excitability is a special fact, but phenomena purely chemical and physical also take place in our bodies, as many of them are explained by weight, porosity, &c. We therefore admit two kinds of forces or phenomena in the living body,—one which is peculiar to organized matter, the other which is common with the physical laws. Our predecessors have attempted all that has been discovered within our time, but they were exclusives—some being chemists, others physiologists, others mechanics, hydrodynamists, &c. What marks our era is not the number of discoveries, but that we take into consideration the facts which have been collected in past ages, without being exclusives, chemists, soldidists, &c. and resuming the labours of our predecessors, we are enabled to improve upon their ideas by the greater lights furnished to us by the sciences of the day. Philosophical doubt is useful to science, but scepticism is fatal to it. Sceptics, although disbelieving the testimony of others, are very credulous as to what they themselves observe, for it is one of the principles of our mind to search for causes of effects produced, and when we meet with a fact to endeavour to trace it up to its origin. Before the cliniques had afforded the advantage of studying diseases from nature, the student was obliged to acquire his information from books, and thus was thrown into practice without having ever seen those affections which he was called upon to treat. But now that the hospitals have been thrown open, and that clinical instruction gives every facility for studying diseases from nature, the student too often thinks that he may dispense with reading; but let me tell you that there can be no good observation without reading, and the number of facts which any one person can observe can never be sufficient to make him acquainted with medicine. On this account, I strongly recommend you to read both the ancient and modern authors.

I shall treat this year in particular of the diseases produced by the alterations of the blood, and then of those of the nervous system.

The alterations of the blood played an important part in the old systems of pathology. The solids were represented by the containing parts, the fluids by the parts contained, and the movements by the vital forces; the ancient Greeks clung to these opinions, being exclusively and successively attached to the doctrines of fluidism, solidism and of the vital forces. Galen proved that these different sects should be united into one, as laid down by Hippocrates. During the seventeenth and eighteenth centuries, the humoral doctrines prevailed, but at the beginning of the present they lost ground; their insufficiency being felt, people were obliged to have recourse to solidism, and among the latter is found Bichat, who, although a solidist, was not an exclusive. Pinel attributed every thing to the alterations of the solids: this doctrine prevailed until about ten years ago, when that of fluidism began to revive, and at that period the alterations of the blood, which to-day are duly recognised, were not admitted. Pinel rejected all the helps of chemistry, but this science, aided by the microscope, is now enlarging the field of our observations.

Every thing comes from the blood, and every thing is carried off by it, and as it is of great importance in physiology, so likewise its importance should be considered in pathology; but until this is confirmed by observation, we will not admit its influence into this science. Many theories have been advanced, but few facts have been established. It is very necessary that we should be well acquainted with the healthy state of the blood, before we can decide upon its alterations. We are still ignorant of the composition of the blood, for the theory which yesterday was looked upon as conclusive and convincing is to-day replaced by a new one. Those who study the microscope see with different eyes; their researches on the blood, like those of the chemists, differing greatly from each other. Another plausible objection which has been raised, is, that the blood which we examine in the cups is no longer the same fluid which circulates in the living vessels: it is not necessary to resolve this problem, for all that is required is to find out if this blood is always the same, and if it is always identical, the alterations which it exhibits, represent those which existed in the living state. History is a prophet come back. Bordeu exclaimed against the chemists, who, by means of their science, explained the vital forces; the systems of chemistry of Kouelle and his contemporaries have been overthrown by that of Lavoisier, and, perhaps, it may come to pass, that the chemical theories which are received to-day will vanish in their turn; for can the chemist pronounce his science to be certain, when so many systems have been subverted? I can show you many facts in pathology, first established by Hippocrates, and which to-day are received as such, proving that the science of medicine contains many positive facts.

The alterations of the blood are of two kinds—in its quantity and in its quality. This latter is the most complex, as it may be changed in its visible properties, in its density, colour, &c.; which changes may be ascertained without the aid of instruments, but by the aid of the microscope we discover others. Alterations in the chemical properties must also be studied; for although we must admit the insufficiency of chemistry at present, yet it furnishes a certain number of positive facts. The blood of an animal affected with anthrax (charbon,) although, with all our means of examination, we can detect no alteration in its composition, injected into a healthy animal will cause its death.

Astruc divided the alterations of the blood into three classes.

In the first class he described the alterations in quantity, which may be increased or diminished. The second class, which he subdivided into three orders, comprised the alterations in its qualities.

1st Order.—Alterations in its physical properties, its consistency, thickening, coagulation, &c.

2d Order.—Alterations of the crassamentum and composition of the blood, by means of foreign particles, pus, miasmata, &c.; or alterations in the principles of the healthy blood, in its serum, fibrin, globules, &c.

3d Order.—Augmentation of its salts, &c.

In his third class he described those alterations resulting from a modification in the motion of the blood, which was either thus affected in its entire mass, or merely in the movements of its globules. This latter question is now attempted to be decided by the microscope.

What forms the subject of our investigation to-day, had also been the study of the ancient teachers.

1. *Alteration of the Blood as to Quantity.*

Any increase or diminution in the quantity of the blood produces a change in its constitution: thus, if it be augmented, the blood becomes richer, and *vice versâ*. We can only tell by analogy and reasoning whether the quantity is increased or diminished; and by observing the phenomena which occur when this change takes place, physiology teaches us that the blood vivifies, gives colour, &c., and when we see all its functions increased, the tissues being more coloured than usual, we admit, by reasoning, that the blood which produces these effects is increased.

This condition constitutes plethora, or general hyperemia. If the sanguineous temperament be exalted, a state of plethora is induced. Formerly, four kinds of plethora were admitted.

1. Plethora ad molem, with respect to the mass of the blood.

2. Ad spatium, related to the space, the vessel being retracted, the blood not being increased.

3. Ad vires, the forces of the circulatory system being irregular.

4. Ad volumen vel spurium, related to the expansibility of the blood, occupying more or less space, depending upon nervous condition, &c.

Some cases occur in which the capillary vessels are more or less dilated, and in which the plethoric state is similated, as in hysteria, and many other nervous diseases; and the same has been found even where the asthenic diathesis has existed; as also after great loss of blood, and in those cases where sanguineous congestions have been formed in different parts.

Is the density of the blood augmented in plethora? It is often very difficult to tell, by the appearance of the blood, whether it is changed in this character. In the plethoric state it presents a large clot, and its density and consistence are considerable; but this state of the blood may be found in a healthy person, and therefore these appearances do not furnish us with any pathognomonic signs. The blood drawn from plethoric persons, does not, in the great majority of cases, present a buffy coat, which is well formed, whitish, resisting, and of half a line in thickness; and if, after phlebotomy, the blood drawn does furnish a well marked buffy coat, it is probable that no plethora exists. Boerhaave thought that the blood of plethoric persons was thicker than that of healthy persons; but of this we have no sufficient proof.

*Influences of Plethora on the Constitution.*

1st. It may modify the symptoms of disease, and should also produce a modification in their treatment.

2d. It may produce several diseases.

3d. It may in itself constitute a disease.

1*st Proposition.*—Plethora was looked upon by the ancients as a state of inflammation, or of general reaction. One of the effects of plethora is to produce local hyperemia or sanguineous congestion; but these latter may increase and become developed with the asthenic state, and will only be removed by such means as will cure the latter—as quinine, &c.

Hæmorrhages are sometimes brought on by a plethoric state. The suppression of the catamenia, by producing plethora, may be the cause of hæmorrhages, although these latter are not always connected with such a condition; as, for instance, apoplexy of the brain, which is seldom dependent upon plethora. I think it is a mistaken notion which supposes plethora as predisposing to inflammation; for, out of thirty cases of pneumonia, two are not produced by, or owing to, a plethoric condition. If out of ten persons exposed to cold, one escape an attack of pneumonia, that one will probably be of a plethoric temperament:

Certain diseases of the skin, such as boils, &c., seem to depend upon a plethoric state; and a cutaneous eruption sometimes appears in those in whom plethora has been induced by a suppression of the menses.

*Second Proposition.*—There are some morbid conditions in which the secretions are increased, which seem to depend upon hyperemia. Plethora, among other causes, produces an augmentation of the serum; giving rise to active dropsies, in which the vessels seem to be too full of blood, and which are relieved by blood-letting: if in living animals we distend the circulatory organs with water, absorption becomes diminished, and the transudation from the vessel increased: some serous exhalations seem to depend upon the quantity of blood in the vessels being increased. Plethoric persons perspire freely and constantly through the skin, and if this perspiration be checked, the skin becomes the seat of a pruritus, and symptoms of fever may show themselves, which will disappear when the perspiration is re-established: the urine of plethoric persons contains a quantity of uric acid, which forms a deposite of red particles, and their blood being very rich, we might suppose that nutrition would be greatly increased but we do not find this to be the case, although the heart is sometimes found hypertrophied. I do not believe, generally speaking, that plethora excludes the formation of tubercles and other morbid productions; but this rule is not without exceptions; if cancer becomes developed in a plethoric person, who is at the same time of a sanguineous temperament, it gradually destroys the state of plethora. Plethora also exercises certain influences over the nervous system in congestions of the brain and of the respiratory apparatus.

*Third Proposition.*—The plethoric state being exaggerated or increased to excess, may in itself constitute a disease in which all the functions become deranged, and a fever is produced. I am of opinion, that plethora increasing to a state of excess, may cause fever in the same way as it may produce derangement of a single function; the fever thus taking place in a plethoric person, always assumes the imflammatory form, and requires bloodletting. This fever may be symptomatic of a phlegmasia of the heart or the vessels of the circulatory system, or may depend upon the over excitement of the organs by the blood, which is too rich, and may be designated hyperemic fever; it is short in its duration, and terminates favourably; the state of excitement being calmed by evacuations preceded by perspirations or by epistaxis, or hæmorrhage from the uterus or hæmorrhoidal veins, the blood by these means being diminished in its quantity or richness. Or it may terminate in another manner, the excitation being determined to a particular organ, which henceforth will constitute the predominant lesion: there are few phlegmasiæ which are not preceded by this inflammatory fever, succeeding or produced by an exaggerated plethora.

*Causes of Plethora.*—It may exist from birth, or become developed without any particular cause. There are some persons who make too much blood, others on the contrary make too little. We are ignorant of the natural cause of plethora: the different periods of life exercise a great influence over plethora: children are exempt from plethora, on account of the activity of their growth; a state of false plethora is common amongst them, on account of the delicateness of their skin, and this condition is styled lymphatisme. When the growth stops at the time of puberty, plethora frequently shows itself, constituting what is called accidental plethora, which may disappear in a short time. Old age seldom falls into a state of plethora; although from certain causes it may exist at this period of life, the plethoric state exists in a direct ratio with the activity of the functions which produce the blood.

*Influence of Digestion.*—The more abundant is the formation of chyle, the more likely is plethora to be produced; and it is frequently met with, associated with a great increased development of the respiratory apparatus. There are two varieties of old men—one who retain the appearance of youth, and whose lungs resemble those of younger men; and the other, who are decrepit, and whose lungs present enlarged cells, and in whom the organ of hematose is imperfect: persons of a nervous temperament generally have a quick pulse, and are not subject to plethora, and from this circumstance we may infer that the rapid circulation of the blood is not a cause of plethora. The palpitations of the heart, which are met with in plethoric persons, are the effect and not the cause of plethora; if the period at which menstruation begins passes over without the appearance of the discharge, plethora is frequently induced: during the whole period of menstruation it may be produced by any suppression of this secretion, and the cessation of the menses may dispose to it, and it may likewise occur during pregnancy. Plethora shows itself in some individuals at the time of spring; and the acute diseases which occur at this period of the year require to be actively combatted by bloodletting.

*Anemia.*

Anemia is that state which is opposed to plethora, and in which the vessels contain a lesser quantity of blood, which is of a deteriorated or impoverished quality. Some persons bear the loss of a large quantity of blood without falling into this state, whilst in others this condition is produced by the loss of a small quantity. Those of a nervous temperament, and particularly women, do not bear the loss of much blood, and having this in our recollection, when treating their diseases we do not carry our bleedings to the same extent as we would in other cases: hospital patients support bleeding better than those in private practice.

*Age considered with respect to bleeding.*—We meet with some cases when we must bleed, without taking into consideration the age of the patient. The state of anemia is easily produced in children under the age of six years, by large bleedings. Robust old men will bear large bleedings; but those whose lungs are, as it were, atrophied, and in whom the process of sanguification is feebly carried on, are easily thrown into this state, and large bleedings in them may be attended with the most fatal results. In such cases, the bleeding, if employed to arrest the inflammation, if pneumonia is the subject, will fail; the bronchial tubes become filled with mucus, and death takes place. This division of old men into these two classes will explain those different results which we find detailed by authors. The more rapid is the abstraction of blood, the more quickly is anemia produced.

In anemia the blood is altered in its physical properties, as well as in quantity. The proportions of water and serum are increased, and those of the fibrine and globules are lessened.

*Causes of Anemia.*—Some persons, without any cause to explain it, produce an impoverished blood, and present a condition of spontaneous anemia, characterized by paleness of the face, and all the symptoms of debility, without being affected with any organic disease, or having suffered the loss of blood. This state is more frequently met with in women than in men. Is there any difference between anemia and chlorosis? This latter affection is now considered as depending upon the uterus. A man who has lost much blood by hemorrhoids, will present all the symptoms of chlorosis. The blood of a chlorotic patient presents the same alterations as that of an anemic person. Anemia produced by an hæmorrhage, is cured by the same remedies as chlorosis. I look upon chlorosis as a state of spontaneous anemia more frequent among women, and met with not only amongst adult unmarried women, but also amongst married ones, and girls of seven years of age. Anemia may be produced accidentally by hæmorrhoidal discharges, prolonged abstinence, diseases of the lungs, and great efforts and expenditure of nervous influence. About twenty years ago, an epidemic anemia broke out amongst those employed in a mine in the south of France, and all those who were attacked by it presented the symptoms of chlorosis; its causes could never be ascertained, and it is the only case on record of the kind. Anemia may be produced by an unwholesome atmosphere; but the affection thus brought on is of an imperfect form.

*Symptoms.*—Anemia exercises a great influence over the intelligence, sensation, and the movements of the muscular system.

*Effects on the Intelligence.*—Delirium sometimes follows large bleedings; the brain being imperfectly excited by impoverished blood, produces disorders of the intelligence, and the delirium thus produced has been well described by Marshall Hall, who mentions thirty cases which were treated by brandy and beef-tea.

A comatose state, putting on the symptoms of inflammation of the brain, may be connected with a state of anemia;—when persons are recovering from an illness which has brought them to the verge of the grave, and after great debility are beginning to retrieve their strength, their intelligence becomes disordered, and a real state of mania becomes developed. [A case was related of a woman recovering from peritonitis, who was effected with delirium during her convalescence, which was prolonged for six months, at the end of which time her forces having returned, her reason again became sound, her blood at this time being produced richer in quality.]

*Effects of Anemia on Sensation.*—In a number of cases the sensibility increases as the blood becomes impoverished; the skin becomes more irritable and sensible; sounds strike painfully on the ear, odours are disagreeable, and light hurts the eyes. These symptoms disappear when the state of anemia ceases. Total abstinence prolonged brings on anemia, with total loss of the sight, preceded by a state in which the sense of vision becomes exalted. This kind of amaurosis is followed by death, and dissection discovers no lesion of the brain, which is merely found paler than natural. [Case cited in which there was loss of sight produced by a fright, which caused a shock to the nervous system.] Vertigo, headach, singing in the ears, &c., are produced by anemia, and in some cases it is impossible to distinguish the symptoms of anemia of the brain from those depending upon hyperemia of that organ. [Case related of a man who had been repeatedly bled for symptoms which were referred to a cerebral congestion; in consequence of this treatment he became very nervous, pale, and sunken; his sight became affected, his digestion deranged, and his functions disordered. By the gradual exhibition of tonics all these symptoms were in time removed.]

*Contractility affected by Anemia.*—Great weakness of the muscular system in chlorotic females is often the first symptom of anemia, and this debility seems to depend upon the blood containing less fibrine. We sometimes meet with cases of convulsions, in which the muscular system is in a state of exaltation. Animals who die of hæmorrhage die convulsed, and in children convulsions are produced by leeches or by chronic disease, and lying-in women are often thus affected after floodings. Young females, who are pale and chlorotic, are often affected with chorea. Subsultus tendinum is another example. These are all proofs that convulsions exist in connection with anemia, or an impoverished condition of the blood.

[Case of tetanus cited, which came on after an hæmorrhage.]—*Lon. Med. Gaz.*

UNIVERSITY COLLEGE HOSPITAL.

*Cases of Simple and Malignant Tumour of the Lower Jaw.*—Ellen H., aged 19, was admitted January 29, 1840, under the care of Mr. Liston. She is a country girl, and has always enjoyed good health. Is quite sane. She did not suffer from toothache previous to the commencement of her present disease. She had no decayed teeth on the right side of the lower jaw. Six years ago she perceived a trifling swelling of the gum, at the end of the jaw; there was a small abscess, and some discharge. The tumour has continued to increase up to the present time; there has always been some slight discharge into the mouth. As the tumour increased, the teeth were removed. She suffered a good deal of pain in the jaw before the tumour commenced; since that time, however, she has experienced little pain, except when she has caught cold. On admission, there is a tumour situated on the right side of the inferior maxilla, extending from the canine tooth to beyond the angle of the jaw. The tumour is the size of a man's fist. It is hard, and evidently of a fibrous character. Not painful on pressure. The glands of the neck not enlarged. Her general health is quite good. The three last molar teeth in the lower jaw have been extracted, aud their situation is occupied by a red mass of the consistence of gum. Catamenia quite regular; bowels open. Mr. Liston considered the tumour to be a good specimen of the fibrous kind, and that therefore it could be removed with safety. He determined to operate.

*Feb.* 7. The operation was performed to-day.

The patient being seated in a chair, Mr. Liston extracted the lateral incisor of the right side; with a strong, straight bistoury he made an incision, commencing a little above the angle of the jaw, continuing under it to opposite the lateral incisor, then turning upwards towards the mouth, terminating about an inch above the chin; the flap of the integument was raised, and keeping the blade of the knife towards the lower jaw, the muscles were separated at that part where the tooth had been extracted, until the fore-finger could be passed into the mouth. The jaw was then partly sawn through with Hey's saw, and the division completed by the cutting pliers. The jaw being then pulled outwards, the tissues in connection with the tumour at its base were dissected off. The tumour was then firmly grasped, and with the whole substance of the bone, depressed, for the purpose of getting at the articulation; in doing this the bone gave way, and the operator, not having sufficient leverage to suppress the jaw well, experienced some little difficulty in separating the temporal muscle from its insertion. The incision had also been made very low, in order to avoid injuring the branches of the portio dura; this, also, increased the difficulty of dividing the upper attachments of the jaw. In a short time the temporal and masseter muscles were separated from the jaw; the articulation was partly opened, when, in more firmly pressing it, the bone again gave way, leaving the condyle still attached. By seizing this portion of the bone with a sequestrum forceps, it was soon removed. The operation lasted eight or nine minutes, and was borne by the patient with great fortitude. The difficulties of the operation were much increased by the attenuation of the tables and the brittleness of the bone, and its having been twice broken, together with the lowness of the incision, which, although it increases the difficulties of the operation, should always be imitated, since it prevents paralysis, in a great measure. On examining the tumour, it was found to extend as far as the canine tooth on the right side, apparently of a fibrous character in that situation. Near the angle the bone was much expanded, and extremely thinned; this expansion and thinness extended as far up as the condyle, where, also, the bone was much thinned, and filled with a brown, glairy fluid. Previous to the operation, it was not suspected that the tumour extended beyond the angle. This was therefore a case showing the necessity of extirpating the bone from its articulation, inasmuch as considerable disease may extend upwards without much altering the external appearance of the bone. Two harelip pins united the wound in the anterior of the chin, with twisted suture. Ligatures were placed on the facial, transverse facial, and two branches of the internal maxillary. The wound was filled with wet lint, and the patient placed in bed. Little blood was lost during the operation. Eight hours after the operation, reaction came on. The wound was united with three points of suture and some strips of insinglass plaster.

8. She has passed a tolerably comfortable night, though complaining of some pain in the head; has slept several hours; not much fever; pulse 90, not very full. Wound appears to be uniting. The pins and two sutures were removed. To have milk and water.

9. Much the same: going on well; wound entirely united; pulse 100; some little fever and thirst.

10. Passed rather a restless night; complains of pain in the head; feels very weak; cheek somewhat flushed; pulse 100; rather full; skin hot; bowels not open: to have an enema. This produced copious evacuation.

22. Has been going on well since last report. The wound is perfectly healed, and there is very little disfigurement of the face. There is some œdematous swelling on the side where the jaw was removed, but the wound inside and outside the face has completely closed. She suffers no pain; her health is good; she has, in fact, recovered, without the occurrence of a single bad symptom. She has for the last week been wearing an apparatus, to supply the deficiency arising from the removed bone.

March 2. She caught a slight cold a few days since, which was relieved by saline medicine.

10. Discharged cured. To return in three weeks, to have a new apparatus for the mouth.

*Case* 2.—John N., aged fifty-one, was admitted March 24, under the care of Mr. Liston. He is a mechanic. Always enjoyed good health, until the occurrence of the present disease. Had suffered from the toothache, and almost all his teeth were rotton. Seventeen weeks ago he suffered great pain in the last molar but one, and this was followed by some swelling of the gum at the base of the last molar. The swelling increased rather inwards than outwards. From this a tumour commenced, which has continued to increase up to the present time.

He has had no discharge of any consequence from the tumour; he suffered pain in it at the commencement of the disease, but it is now free from pain.

On admission his general health was good. He had a tumour about the size of two fists, extending from above the angle of the lower jaw, on the right side, to near the symphisis, on the same side; proceeding upwards, and becoming moulded on the upper jaw, it extended into the mouth.

The tumour is soft and elastic, but not ulcerated on any part, neither is it liable to bleed; it is unaccompanied by discharge.

*Malignant Tumour of the Lower Jaw.*

James S. was admitted March 26. He had been some years ago accustomed to smoke a short tobacco-pipe, more particularly on the left side of the mouth; this produced some ulceration of the lip, while increasing cancer was the result. He suffered from this for ten years, at the end of which time a portion of the lip was removed, together with the disease. At this time, a year ago, there was a slight swelling underneath the chin, and this has gradually increased up to the present time.

On admission he had a large tumour situated under the centre of the chin, firm and hard, somewhat elastic towards its apex, and of the size of the fist. It had at the apex an ulcerated point, of the size of a sixpence, which bled occasionally when touched. The tumour is firmly adherent to the chin, and the bone seems somewhat enlarged. The skin over it is discoloured, reddened, and firmly adherent. The tumour is very painful at all times, more particularly at night, and when touched. Mr. Liston refused to operate on this case.

In some clinical remarks on the two latter cases, he observed that they afforded the opportunity of showing malignant cases of tumour of the lower jaw, and contrasted well with those cases which had been previously operated upon in the hospital. In the second case the tumour had grown exceedingly rapid, having commenced only seventeen weeks ago. The skin covering it was firmly adherent to it, and ulcerated in several places. Within the mouth was a large fungous growth, which bled when it was touched; and from the history of the case it appeared that the disease commenced in the soft parts.

There could be no doubt that tumours of a much more doubtful nature might be removed from the lower than the upper jaw. In the upper jaw, if the tumour were at all "mali moris"—it might commence under, at an early period, extend into the sphenoidal or ethmoidal cells, and could not be extirpated with safety to the life of the patient. In the lower jaw, where the tumour was confined to the bone, it could be entirely removed, the bone being sawn through wide of the disease, and removed at the articulation. It was not the size of the tumour in the case under consideration, but its character, which determined him from operating. From the skin being so extensively involved in the disease, it would be hardly possible to remove the whole; at any rate, the operation would be such that the patient would probably sink under it, though the disease could be completely extirpated. It could not be expected that the patient would remain free from disease. Under these circumstances, any operation, the slightest, could not be justified.

In the second case, the disease was of a different nature. Its history showed it to be cancer of the lymphatic glands beneath the jaw. The man had originally a cancerous affection of the lip; this had been removed, the lip healed, the cicatrix was quite firm, and there appeared but little disease in the part; the affection, however, had exhibited itself in the lymphatic glands. This was by no means an uncommon occurrence. Patients affected with cancerous diseases had operations performed for their removal; the wound healed, the patients appeared quite well, and the cicatrix remained firm. This state might continue for two or three years, but during that time it would seem as though the seeds of the same disease were laying dormant in the lymphatic glands leading from the previously affected part; and, perhaps, after the lapse of some years, the patient would perish from the development of cancer in these glands. The glands had become affected in this case.

---

HOSPITAL BEAUJON.

*Fall from a height of thirty feet.—Extensive injury of the vertebral column.—Application of the Trephine.—Death.*—On the evening of the 6th of March, 1840, was brought into the Wards of M. Langier, at the Hospital Beaujon, a man aged 25, who, together with a fellow-workman, had fallen from a third story on his back.

At the morning visit, on the 7th, he gave the following account of his accident:—The plank on which he had been standing had given way, and he, with his comrade, was precipitated from a height of thirty feet. During his fall he turned over once or twice, and struck against some projecting pieces of scaffolding, and finally fell on a heap of stones on his back. He became insensible, his comrade had fallen upon him, and had escaped with simply a severe sprain of one of his ankles.

The patient was laying on his back, with the usual symptoms of injury of the spinal marrow; total loss of sensibility and movement of the lower extremities, extending upwards as far as the umbilicus; retention of urine and fæces; pain along the spine; pulse full, rather quick; countenance anxious; pain in epigastric region, on breathing deeply; respiration rather quicker than natural; thirst, &c. On passing the finger along the spine, a depression, with slight sensation of crepitation, was felt, apparently, between the seventh and eighth dorsal vertebræ. The skin over the part and back generally showed no signs of contusion.

Venesection to sixteen ounces; quietude; position on the back to be retained, and urine to be drawn off twice a-day.

On the 11th, five days from the accident, the symptoms were in no degree alleviated. Loss of sensibility and motion; retention of urine and fæces, as before. The respiration still continued hurried, and the buttocks and heels were inflamed, and a slough had begun to form on the sacrum, although no passage of fæcal matter had taken place; the abdomen was soft and tender, and not distended with gases.

M. Langier determined to apply the trephine, and by making an opening into the vertebral canal, to attempt to remove the pressure on the marrow, whether depending on depressed bone, or effused fluid. From an external examination, it seemed impossible to ascertain the actual extent of the injury, or the immediate cause of the symptoms; but M. L. judged the operation advisable, as the "remedium anceps" in a case otherwise mortal.

*Operation.*—The patient being placed on the side, almost reclining on the face, an incision, from three to four inches, was made along the mesial line, opposite the depressed spot, and the dorsal muscles were cut through, and detached on each side from the spinous processes, which were thus completely exposed. The spinous process of the ninth dorsal vertebra was found to be broken off, and was easily dissected out, bringing with it a portion of the posterior arch: thus, a small aperture was made in the vertebral canal, but it was not judged sufficient; a small trephine was then placed on the base of the spinous process of the vertebra below, or the eighth, and the circular portion of bone removed; an opening, large enough to admit the fore finger, was thus formed, and the medula, covered by its theca, exposed, which, as far as could be ascertained, appeared uninjured. No depressed portion of the bone could be felt, but some blood escaped from the canal. It being evident that nothing further could be done, the wound was simply dressed with a piece of linen, smeared with cerate, and the patient was again laid on his back, so as to facilitate the escape of fluids, if any such existed in the canal.

The operation, which, according to some authors, is extremely painful and difficult, was easily and rapidly performed; the sufferings appeared slight, and little blood was lost, no vessel requiring ligature.

12. At the morning visit, we found the patient laying on his right side; he told us that about two hours after the operation, his respiration had become more free, and the pain caused by inspiration less; but that, in the middle of the day, he had been seized with a severe convulsive attack of coughing, which had lasted all night; towards the evening this had become so distressing, with pain running round the chest to the wound, that mustard poultices were applied to the legs, and had produced some relief; their presence had excited a sensation of warmth in the legs, but no smarting or pricking was felt; also, towards evening, the introduction of the catheter having been neglected, the pain and weight in the pubic region, from distention of the bladder, with desire to void the urine, had been felt, but the subsequent introduction of the catheter had excited no sensation; he had had some disturbed sleep during the night. This morning his state is—face flushed; skin hot, but moist, tongue dry, furred, with redness at the tip; pulse rapid, 128 pulsations, depressible; no rigours; respiration somewhat easier and freer; abdomen slightly inflated; no passage of fæcal matter, but of wind expelled with force; urine high-coloured, but neither turbid nor ammoniacal.

On first inquiry into the state of sensibility in the lower extremities, some amelioration was thought to have taken place, but, after repeated and careful investigation, this turned out to be merely the effect of over-anxiety on the part of the patient, who deceived himself into the belief that he had recovered somewhat the sensibility of the parts: power of motion null, as before; the extremities were warm, and a slight degree of redness had been produced by the mustard poultices.

Fifteen leeches to the back, in the neighborhood of the wound; to drink freely, and some broth to be given.

13. We found the patient this morning in a great state of excitement; face turgid; hurried respiration; quick, small, wiry pulse, 130 pulsations; great thirst; pupils dilated; skin hot, dry; voice low, but distinct: the account given was, that he had again been attacked with a severe fit of coughing, which continued through the night, and was attended with sharp dragging pains over the chest, radiating from the wound; he had also experienced a sharp attack of rigors, with vomiting and headach; some fæcal matter had passed involuntarily, and a second attack of rigors, which came on in the evening, he attributed to exposure whilst the nurses were cleaning him; just before the visit this morning he had another involuntary discharge of dark-coloured, fœtid fæces; the buttock was not only inflamed, but a slough had formed; the wound looked well; considerable quantities of sero-sanguinolent fluid had escaped from it.

14. General appearance and symptoms much worse; respiration more hurried and oppressed, diaphragmatical; 130 to 140 pulsations; had a severe attack of rigors yesterday, and another this morning; has been delirious and agitated during the night, tossing his arms about; has a severe hacking cough, with a kind of a hiccup, or convulsive action of diaphragm, with little sound; belly soft; the meteorism, described by authors as a constant symptom, does not exist, nor is the urine ammoniacal.

These symptoms increased during this and the following day, on which he died, at nine in the evening, five days from the operation.

*Post-mortem Appearances.*—Fracture, without displacement, but comminuted, of the posterior arches of four dorsal vertebræ, from the sixth to the ninth inclusive; or, rather, a double fracture running on each side, at the junction of the articular processes with the roots of the spinous, which were thus completely separated. The spinous process of the seventh, as before mentioned, had been removed in the operation. The perforation made by the trephine in the base of the eighth, occupied exactly the space between the oblique processes, but in no way interested their articulations. No depression of the posterior arches existed. On laying bare the medulla, with its theca, considerable quantities of dark, coagulated blood, mixed with pus, was found laying on the latter, between it and the posterior arch of the canal, extending for an inch or more above and below the perforated spot. The vessels of the theca were gorged with blood. The medulla was found completely disorganized, transformed into a creamy substance to a considerable extent above and below; and opposite the eighth dorsal vertebra its continuity was completely destroyed. Vessels of the medulla also gorged, but no extravasation of blood between it and its theca. The body of the eighth dorsal vertebra was broken into fragments, some projecting into the canal and compressing the marrow. The body of the seventh was also fractured longitudinally, simply a fissure without displacement; the intermediate cartilage had nearly disappeared. The ninth rib on the left side was also fractured, at the distance of an inch from its articulation with the spine. There was no wound of the pleura, but the pleural sac of the same side was found inflamed, with considerable sero-sanguinolent effusion, and the left lung inflamed and hepatised.—*Lon. Lan. April*, 1840.

---

*Case of Prolapsus Ani, Cured by Actual Cautery.* By John Scott, Esq.—Nuttoo Milarsett, goldsmith, was brought to the Poor Dispensary on the 8th of November, 1837. He stated that he had been for some years subject to a prolapsus of the rectum, coming down with every stool, yet easily reducible by the hand after it; that five days ago, the protruded part had become much larger, and since then he had not been able to return it.

The tumor was about five inches in length, and about twelve in circumference, bulging over the verge of the anus, and conical towards the extremity. Considerable ulceration had taken place on the surface, which, in general, was of a darkish red appearance, and in some parts covered with a superficial black slough.

His body was amaciated, and his constitution apparently much debilitated by the disease. On admission, his bowels were confined, and he complained of difficulty in passing his urine, for which he was ordered some castor oil and nitric æther.

For some days, cold lotions were applied to the tumor, and his bowels kept open with castor oil. Leeches, also, were several times applied to the verge of the anus, and once to the tumor, when it appeared swollen and inflamed; a bitter infusion was given to support the strength. As the difficulty in passing his urine occurred, with occasional pain over the pubis, a saline mixture was exhibited, which relieved this, and kept the bowels free.

All attempts to reduce the tumor were ineffectual; and as it did not seem to be at all diminished in size after thirteen days, it became necessary to try other means to relieve the man from a disease which incapacitated him from earning his livelihood, and was gradually exhausting his strength.

On the 21st of November, a fire spatula, heated to as intense a read heat as could be managed, was passed rather quickly over the whole surface of the tumor. In the cases referred to, it is said, the iron was brought to a white heat, but this the means at hand did not admit of. The pain during the operation seemed greatly less than was anticipated; the patient rested on his knees and elbows, so as to present the buttocks, without being held. Simple dressing was applied after the operation, and no unpleasant symptoms followed; the only new one complained of was a feeling of straining at stool. The difficulty in passing urine continued with the pain over the pubis; but both were relieved, and his bowels kept free by the saline mixture.

The tumor at first appeared shrunk, but could not be reduced; the ulcers on the surface healed, and there was a considerable watery discharge for a day or two from the surface. There having been no success, and the patient having suffered so little from a first attempt, the cautery was again applied on the 20th, and to do it more effectually, two irons were used, the second being taken when the first became black. On this occasion there was a striking proof of how much the appearance of severity in the remedy differed from the reality of it; for, after both irons had been used, the man pointed to a part of the tumor which had been less cauterised than the rest, and desired that the iron might be re-applied, probably from the effect the cautery had in reducing the irritation arising from the large exposed surface.

Little or no change was produced on the tumor by this second attempt, but still no unpleasant symptoms followed; the patient continued in every way much the same till the 14th of December, when the cautery was again applied. One iron only was used this time, but more slowly and forcibly; on this occasion, too, the man applied for a re-application of the iron. A greater change immediately followed this application than either of the former; the tumor became more shrunk, both in length and diameter, than it had before done, and continued so. On the 16th the man desired leave to go home and return, which he did; and on the 17th he again went out, but did not return. Up to the 6th of January he has had no return of the prolapsus.

The above case shows with how much safety so formidable a remedy as actual cautery may be applied to the protruded gut, and may induce others to attempt to cure, in the same manner, this loathsome disease, which is not uncommon, I believe, in this country.—*Trans. of Med. and Phys. Society of Bombay*, vol. ii.

